# Efficient TCT-catalyzed Synthesis of 1,5-Benzodiazepine Derivatives under Mild Conditions

**DOI:** 10.3390/molecules13092313

**Published:** 2008-09-25

**Authors:** Chun-Wei Kuo, Chun-Chao Wang, Veerababurao Kavala, Ching-Fa Yao

**Affiliations:** Department of Chemistry, National Taiwan Normal University, 88, Section 4, Tingchow Road, Taipei 116, Taiwan, ROC

**Keywords:** Benzodiazepines, 2,4,6-trichloro-1,3,5-triazine, enolizable ketones, *o*-phenylenediamines

## Abstract

2,4,6-Trichloro-1,3,5-triazine (TCT) efficiently catalyzed the condensation reactions between 1,2-diamines and various enolizable ketones to afford 1,5-benzodiazepines in good to excellent yields. Simple and mild reaction conditions, the use of a cheap catalyst and easy workup and isolation are notable features of this method.

## Introduction

Benzodiazepines and its derivatives constitute an important class of heterocyclic compounds which possess a wide range of therapeutic and pharmacological properties. Derivatives of benzodiazepines are widely used as anticonvulsant, antianxiety, analgesic, sedative, antidepressive, and hypnotic agents [1,2]. In the last decade, the area of biological interest of 1,5-benzodiazepines has been extended to several diseases such as cancer, viral infection and cardiovascular disorders [3,4]. In addition, 1,5-benzodiazepines are key intermediates for the synthesis of various fused ring systems such as triazolo-, oxadiazolo-, oxazino- or furanobenzodiazepines [5,6,7,8]. Besides, benzodiazepine derivatives are also of commercial importance as dyes for acrylic fibers in photography [9]. Owing to their versatile applications various methods for the synthesis of benzodiazepines have been reported in the literature. These include condensation reactions of *o*-phenylenediamines with α,β-unsaturated carbonyl compounds [10], with ketones in the presence of BF_3_·Et_2_O, NaBH_4_ , polyphosphoric acid or SiO_2_ , MgO/POCl_3_ , Yb(OTf)_3_, Al_2_O_3_/P_2_O_5_ or AcOH under microwave conditions, Amberlyst-15 in the ionic liquid 1-butyl-3-methylimidazolium bromide ([bmim]Br), CeCl_3_·7H_2_O/NaI supported on silica gel, InBr_3_ , Sc(OTf)_3_ , sulfated zirconia, InCl_3_ , CAN, ZnCl_2_ under thermal conditions, AgNO_3._ [11,12,13,14,15,16,17,18,19,20,21,22,23,24,25,26]. These reactions also occur with various catalysts under solvent free conditions [27,28,29,30]. Nevertheless, many of these methods suffer from shortcomings such as long reaction times, harsh reaction conditions, low product yields, occurrence of several side products and difficulties in recovery of the products. Moreover, some of the reagents employed are very expensive. Consequently, the search continues for better catalysts in terms of operational simplicity and economic viability to synthesize 1,5-benzodiazepines.

In recent years, 2,4.6-trichloro-1,3,5-triazine (TCT) has received considerable attention due to its commercial availability and efficient delivery of anhydrous HCl in reaction media. It is inexpensive, and has been found to be versatile in functional group transformations such as conversions of alcohols to alkyl chlorides, oxidations of sulfides to sulfoxides, oxidative couplings of thiols and selenols, cleavage of thioacetals, etc. [31,32,33,34,35,36]. TCT reacts with ‘incipient’ moisture and produces three moles of HCl and cyanuric acid as a by-product (removable by simple washing with water). The HCl generated *in situ* acts as a protic acid, activates the carbonyl oxygen to promote the condensation to give the products [37]. In continuation to our efforts for the development of simple and novel methods for the synthesis of different heterocyclics [38,39,40,41,42,43,44], we report herein a simple and efficient method for the synthesis of 1,5-benzodiazepines using TCT as catalyst. While this paper was under peer review, Khodaei *et al*. [45] reported a similar procedure, differing from ours in the preferred solvent – CH_3_CN *vs*. MeOH – and the amount of solvent required, 10 mol % *vs*. 4 mol %; the yields were similar although their reported reaction times were shorter, possibly due to the higher catalyst load used.

## Results and Discussion

**Scheme 1:**
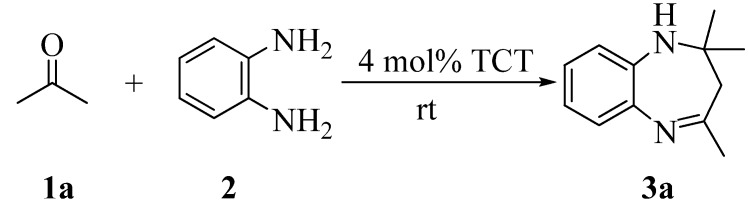
Synthesis of 1,5-benzodiazepines.

In the first instance, *o*-phenylenediamine (1 equiv.) and acetone (2.5 equiv.) were stirred at ambient temperature in dichloromethane with 4 mol% of TCT (Scheme 1). The reaction was complete within 5.5 h. After screening various solvents like methanol, ethanol, isopropanol, ethyl acetate and acetonitrile, we found that the reaction proceeds well in polar solvents, giving slight variations in reaction time and that methanol was the best choice for this reaction (Table 1).

We assume that in the reaction medium TCT generates anhydrous HCl, which would be the active catalyst. When a similar reaction was performed using 10 mol% of aqueous HCl, the reaction took longer time (24 h) for completion, whereas the same conversion was achieved in 1 h with anhydrous 10% HCl in methanol. This further supports the proposed *in-situ* generation of anhydrous HCl in the reaction as the source of the catalytic action.

**Table 1:** Solvent effects in the reaction.

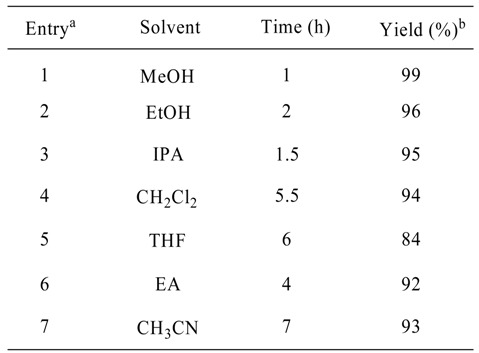

^a^ The reaction was performed with acetone (2.5 mmol) and diamine (1 mmol) in 1 mL of solvent catalyzed by TCT.

^b^ Isolated yield.

**Scheme 2:**
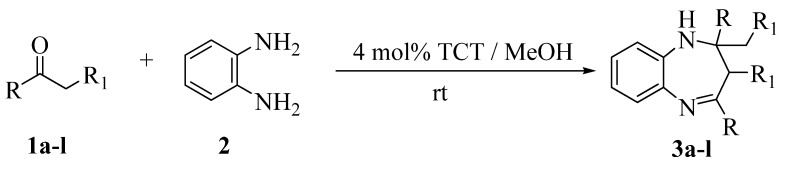
Reaction of *o*-phenylenediamines with various ketones in the presence of 4 mol % of TCT.

Under the optimized conditions, aliphatic ketones such as acetone reacted with 1,2-phenylenediamine in methanol (Scheme 2) to form the corresponding benzodiazepine in excellent yield (Table 2, entry 1). However, the reaction was sluggish with the substrates 2-butanone and 3-pentanone (Table 2, entries 2 and 3), resulting in poor yields. This may be due to the steric hindrance of a methyl group in the proximity of the carbonyl carbon. Alicyclic ketones such as cyclopentanone, cyclohexanone and cycloheptanone (Table 2, entries 4-6) gave excellent yields of products. With the present methodology, aromatic ketones such as acetophenone (Table 2, entry 7) and substituted acetophenones with both electron-donating and withdrawing groups generally produced the corresponding benzodiazepines in good to excellent yields, with the latter performing somewhat better. Thus, for example, an acetophenone bearing a OMe electron-releasing group such as 4-methoxy- acetophenone (Table 2, entry 8) resulted in a poorer yield after a longer period of time, whereas an acetophenone possessing a NO_2_ electron-withdrawing group, such as 4-nitroacetophenone (Table 2, entry 11) underwent a smooth reaction to afford a good yield of the corresponding product **3k**.

**Table 2:** The results of the reaction of *o*-phenylenediamines with various ketones.

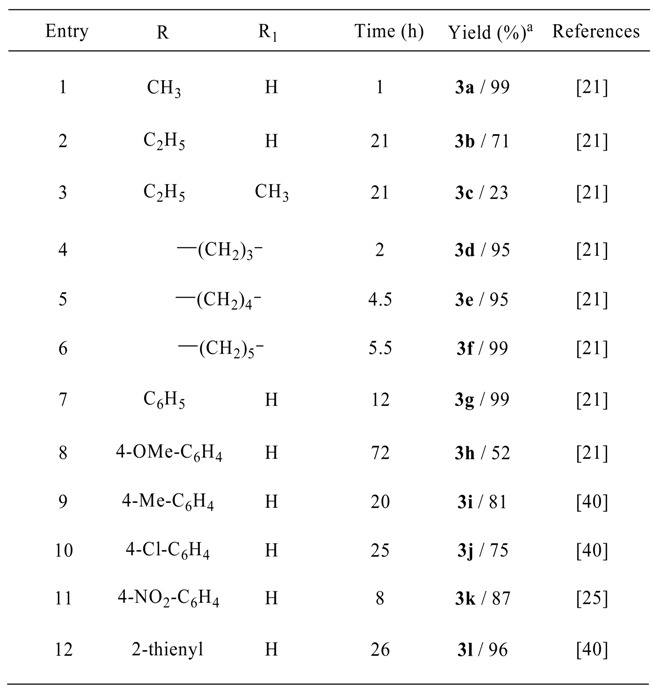

^a^ Isolated yields.

**Scheme 3:**
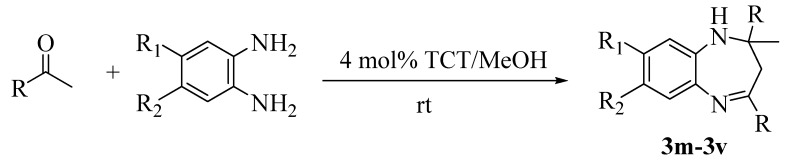
Reaction of various *o*-phenylenediamines with ketones in the presence of 4 mol % of TCT.

Having successfully performed the reactions of 1,2-phenylenediamine with a wide range of ketones, we focused our attention on examining the reactions of various ketones and structurally diverse diamines (Scheme 3). The results revealed that both mono- and disubstituted phenylenediamines reacted with ketones to produce the corresponding benzodiazepines in excellent yields. Diamines bearing substituents with various electronic effects reacted with acetone with equal ease (Table 3, entries 1, 3, 5 and 8). The reactions of aromatic ketones with monosubstituted diamines furnished the corresponding benzodiazepines in shorter times (Table 3, entries 2 and 4), whereas disubstituted diamines took relatively longer times to afford good yields of products (Table 3, entries 6, 7 and 9). All the monosubstituted diamines gave 1:1 mixtures of regioisomers. The results are summarized in Table 3.

**Table 3:** The results of the reaction of various *o*-phenylenediamines with ketones.

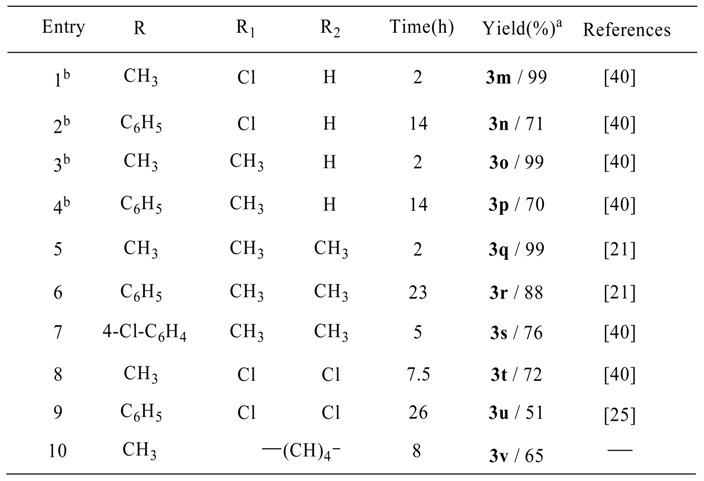

^a^ Isolated yields.

^b^ 1:1 mixture of two regioisomers.

**Scheme 4:**
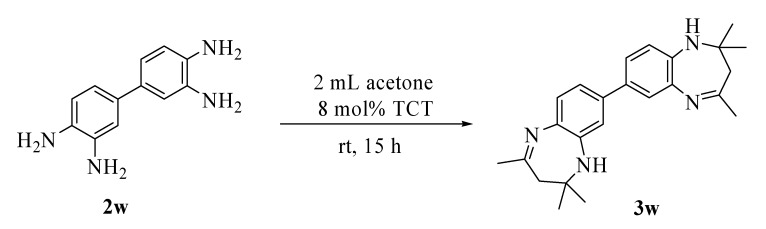
Reaction of bisdiamine with acetone in the presence of TCT.

Finally, the reaction of acetone with a bisdiamine in the presence of 8 mol % TCT gave the corresponding bisbenzodiazepine in moderate yield (Scheme 4). The product **3w** thus obtained proved unstable in solution and readily decomposed in methanol when it was subjected to crystallization.

## Conclusions

In summary, we have disclosed an efficient and economic method for the synthesis of 1,5-benzodiazepines. We also demonstrated the electronic effects on the reaction of various substitutions on the ketone and the diamine participants. Electron withdrawing groups like the (NO_2_) group stimulate the reaction rate, whereas electron releasing groups reduce the reactivity of the ketone. Simple workup and easy isolation under mild reaction conditions are the best features of the present methodology.

## Experimental

### General

All reagents and chemicals were purchased from Sigma-Aldrich Chemical Company, Acros organics and Merck and were used as received. Analytical thin layer chromatography was performed with E. Merck silica gel 60F glass plates and flash chromatography by the use of E. Merck silica gel 60 (230–400 mesh). ^1^H-NMR and ^13^C-NMR spectra were recorded at 400 and 100 MHz, respectively, on a Bruker Avance EX 400 FT-NMR instrument. Chloroform-*d* was used as the solvent and TMS (*δ* = 0.00 ppm) as an internal standard. Chemical shift values are reported in ppm relative to TMS in delta (*δ*) units. Multiplicities are recorded as s (singlet), d (doublet), t (triplet), q (quartet), dd (doublet of doublet), br (broadened), m (multiplet). Coupling constants (*J*) are expressed in Hz. MS and HRMS were measured on JEOL JMS-D300 and JEOL JMS-HX110 spectrometers, respectively.

### General procedure for the preparation of 1,5-benzodiazepines

To a stirred solution of *o*-phenylenediamine (1 mmol) in MeOH (1 mL), a ketone (2.5 mmol) and 4 mol% TCT were added. The reaction mixture was stirred at room temperature until the reaction was complete, as judged by TLC analysis. The reaction mixture was then concentrated and washed with water to give the crude product, which was further purified by flash chromatography on silica gel (eluent: hexane-EtOAc= 5:1).

*2,2,4-Trimethyl-2,3-dihydro-1H-benzo[b][1,4]diazepine* (**3a**): ^1^H-NMR *δ* 7.14–7.12 (m, 1H), 7.00–6.96 (m, 2H), 6.74–6.72 (m, 1H), 2.97 (br s, 1H, NH), 2.37 (s, 3H), 2.23 (s, 2H), 1.35 (s, 6H); ^13^C-NMR *δ* 172.4, 140.8, 137.9, 126.9, 125.5, 122.1, 121.7, 68.3, 45.2, 30.5, 29.9; MS (EI) *m/z* (relative intensity) 188 (M^+^, 38), 173 (100), 133 (39), 132 (50); HRMS (EI) *m/z* calcd. for C_12_H_16_N_2_ (M^+^) 188.1314, found 188.1314.

*2,2-Dimethyl-2-methyl-2,3-dihydro-1H-1,5-benzodiazepine* (**3b**): ^1^H-NMR *δ* 7.16–7.14 (m, 1H), 6.99–6.95 (m, 2H), 6.73–6.71 (m, 1H) 3.10 (br s, 1H, NH), 2.61–2.57 (m, 2H), 2.22 (d, *J* = 12.9 Hz, 1H), 2.14 (d, *J* = 12.9 Hz, 1H), 1.63 (m, 2H), 1.24 (m, 5H), 0.95 (t, *J* = 7.5 Hz, 1H); ^13^C-NMR *δ* 175.9, 140.7, 137.9, 127.1, 125.4, 121.8, 121.7, 70.7, 42.1, 35.7, 26.9, 10.6, 10.6, 8.5; MS (EI) *m/z* (relative intensity) 216 (M^+^, 19), 201 (20), 187 (100), 145 (18); HRMS (EI) *m/z* calcd. for C_14_H_20_N_2_ (M^+^) 216.1627, found 216.1623.

*2,2,4-Triethyl-3-methyl-2,3-dihydro-1H-1,5-benzodiazepine* (**3c**): ^1^H-NMR *δ* 7.35 (dd, 1H, *J* = 7.9, 1.1 Hz), 6.97 (t, *J* = 8.1 Hz, 1H), 6.74 (t, *J* = 7.1 Hz, 1H), 6.62 (dd, *J* = 8.0, 0.5 Hz, 1H), 3.86 (br s, 1H, NH), 2.85 (q, *J* = 7.0 Hz, 1H), 2.60–2.49 (m, 2H), 1.61–1.51 (m, 2H), 1.37 (q, *J* = 7.4 Hz, 2H), 1.24 (t, *J* = 7.4 Hz, 3H), 0.96–0.88 (m, 6H), 0.79 (t, *J* = 7.3 Hz, 3H); ^13^C-NMR *δ* 173.6, 139.0, 132.8, 132.2, 126.7, 117.9, 117.5, 60.3, 46.1, 35.7, 28.4, 28.0, 12.3, 11.5, 7.8, 7.3; MS (EI) *m/z* (relative intensity) 244 (M^+^, 14), 216 (12), 215 (100), 147 (23); HRMS (EI) *m/z* calcd. for C_16_H_24_N_2_ (M^+^) 244.1940, found 244.1940.

*2,3,9,10a-Tetrahydro-1H-spiro[benzo[b]cyclopenta[e][1,4]diazepine-10,1’-cyclopentane]* (**3d**): ^1^H- NMR *δ* 7.32 (dd, *J* = 1.2, 1.1 Hz, 1H), 6.99–6.76 (m, 2H), 6.57 (dd, *J* = 1.0, 1.0 Hz, 1H), 3.98 (s, 1H), 2.77 (t, *J* = 9.0 Hz, 1H), 2.63–2.59 (m, 2H), 2.15–2.06 (m, 1H), 2.00–1.92 (m, 1H), 1.87–1.56 (m, 9 H), 1.48–1.42 (m, 1H); ^13^C-NMR *δ* 178.2, 139.3, 134.1, 132.4, 127.1, 119.5, 118.9, 67.5, 54.4, 39.5, 38.7, 33.6, 29.1, 24.4, 24.2, 23.6; MS (EI) *m/z* (relative intensity) 240 (M^+^ , 39), 211 (87), 183 (42), 145 (41), 132 (100); HRMS (EI) *m/z* calcd. for C_16_H_20_N_2_ (M^+^) 240.1626, found 240.1629.

*2',3',4',10'-Tetrahydro-1'H-spiro[cyclohexane-1,11'-dibenzo[b,e][1,4]diazepine]* (**3e**): ^1^H-NMR *δ* 7.29–7.26 (m, 1H), 6.99–6.92 (m, 2H), 6.70 (d, *J* = 1.4 Hz, 1H), 3.78 (br s, 1H, NH), 2.59 (t, *J* = 6.6 Hz, 2H), 2.4–2.36 (m, 1H), 1.86–1.18 (m, 16H); ^13^C-NMR *δ* 176.2, 138.6, 138.3, 129.4, 126.0, 121.2, 121.1, 51.9, 41.9, 40.9, 40.6, 34.2, 33.0, 27.2, 26.9, 26.7, 25.3, 24.2, 21.9; MS (EI) *m/z* (relative intensity) 268 (M^+^, 39), 225 (100), 145 (53), 132 (39); HRMS (EI) *m/z* calcd. for C_18_H_24_N_2_ (M^+^) 268.1940, found 268.1945.

*7,8,9,10,10a,12-Hexahydro-6H-spiro[benzo[b]cyclohepta[e][1,4]diazepine-11,1'-cycloheptane]* (**3f**): ^1^H-NMR *δ* 7.21–7.19 (m, 1H), 7.01–6.96 (m, 2H), 6.73–6.71 (m, 1H), 3.59 (br s, 1H, NH), 2.80–2.64 (m, 2H), 2.36–2.32 (m, 1H), 2.05–0.99 (m, 20H); ^13^C-NMR *δ* 179.9, 137.1, 127.1, 125.5, 121.8, 121.6, 54.0, 40.6, 38.3, 37.9, 30.2, 29.8, 29.7, 29.0, 28.3, 26.2, 23.2, 22.4; MS (EI) *m/z* (relative intensity) 296 (M^+^, 39), 239 (100), 145 (29), 132 (35); HRMS (EI) *m/z* calcd. for C_20_H_28_N_2_ (M^+^) 296.2253, found 296.2250.

*2-Methyl-2,4-diphenyl-2,3-dihydro-1H-benzo[b][1,4]diazepine* (**3g**): ^1^H-NMR *δ* 7.59–7.57 (m, 4H,), 7.31–7.16 (m, 7H), 7.05–7.03 (m, 2H), 6.83 (d, *J* = 1.7 Hz, 1H), 3.50 (br s, 1H, NH), 3.12 (d, *J* = 13.2 Hz, 1H), 2.95 (d, *J* = 13.2 Hz, 1H), 1.74 (s, 3H); ^13^C-NMR *δ* 167.6, 147.6, 140.1, 139.6, 138.0, 129.7, 128.6, 128.3, 128.0, 127.0, 127.0, 126.3, 125.4, 121.6, 121.4, 73.6, 43.0, 29.8; MS (EI) *m/z* (relative intensity) 312 (M^+^, 24), 297 (25), 235 (23), 194 (100); HRMS (EI) *m/z* calcd. for C_22_H_20_N_2_ (M^+^) 312.1627, found 312.1632.

*2,3-Dihydro-2-methyl-2,4-di(4'-methoxyphenyl)-1H-1,5-benzodiazepine* (**3h**): ^1^H-NMR *δ* 7.59 (d, *J* = 8.7 Hz, 2H), 7.51 (d, *J* = 8.7 Hz, 2H), 7.29 (t, *J* = 5.2 Hz, 1H), 7.03 (t, *J* = 4.4 Hz, 2H), 6.81–6.75 (m, 5H), 3.78 (s, 3H), 3.74 (s, 3H), 3.41 (br s, 1H, NH), 3.03 (d, *J* = 13.2 Hz, 1H), 2.90 (d, *J* = 13.2 Hz, 1H), 171 (s, 3H); ^13^C-NMR *δ* 167.1, 161.0, 158.5, 140.6, 140.1, 138.0, 132.3, 128.8, 128.2, 126.5, 125.8, 121.7, 121.5, 113.5, 113.3, 73.3, 55.3, 55.2, 42.8, 29.7; MS (EI) *m/z* (relative intensity) 372 (M^+^, 9), 357 (8), 225 (23), 224 (100), 133 (14); HRMS (EI) *m/z* calcd. for C_24_H_24_N_2_O_2_ (M^+^) 372.1838, found 372.1832.

*2,3-Dihydro-2-methyl-2,4-di(4'-methoxyphenyl)-1H-1,5-benzodiazepine* (**3i**): ^1^H-NMR *δ* 7.53 (d, *J* = 8.0 Hz, 2H), 7.44 (d, *J* = 8.0 Hz, 2H), 7.31–7.28 (m, 1H), 7.05–6.99 (m, 6H), 6.76–6.74 (m, 1H), 3.44 (br s, 1H, NH), 3.03 (d, *J* = 13.2 Hz, 1H), 2.92 (d, *J* = 13.2 Hz, 1H), 2.30 (s, 3H), 2.27 (s, 3H), 1.67 (s, 3H); ^13^C-NMR *δ* 167.3, 144.9, 140.2, 139.8, 138.1, 136.9, 136.5, 129.1, 128.9, 128.7, 128.4, 127.1, 126.0, 125.1, 121.5, 121.3, 73.2, 42.7, 29.7, 21.2, 20.8; MS (EI) *m/z* (relative intensity) 340 (M^+^, 9), 325 (7), 209 (40), 208 (100), 207 (14), 133 (15), 117 (55), 92 (26), 91 (18), 65 (10); HRMS (EI) *m/z* calcd. for C_24_H_24_N_2_ (M^+^) 340.1940, found 340.1944.

*2-Methyl-2,4-di(4-chlorophenyl)-2,3-dihydro-1H-1,5-benzodiazepine* (**3j**): ^1^H-NMR *δ* 7.52–7.46 (m, 4H), 7.28 (d, *J* = 2.0 Hz, 1H), 7.21–7.18 (m, 4H), 7.08–7.04 (m, 2H), 6.82 (d, *J* = 8.8 Hz, 1H), 3.42 (br s, 1H, NH), 3.08–3.04 (d, *J* = 13.2 Hz, 1H), 2.87 (d, *J* = 13.2 Hz, 1H), 1.72 (s, 3H); ^13^C-NMR *δ* 166.0, 145.8, 139.9, 137.7, 137.6, 136.0, 133.0, 128.6, 128.3, 128.2, 127.0, 126.6, 122.0, 121.5, 73.4, 42.9, 29.7; MS (EI) *m/z* (relative intensity) 380 (M^+^, 4), 365 (5), 231 (18), 230 (40), 229 (60), 228 (100), 193 (12), 137 (12), 133 (11); HRMS (EI) *m/z* calcd. for C_22_H_18_N_2_Cl_2_ (M^+^) 380.0847, found 380.0849.

*2,4-bis(4-Nitrophenyl)-2,3-dihydro-1H-benzo[b][1,4]diazepine* (**3k**): yellow solid; m.p. 152*—*154°C; ^1^H-NMR *δ* 8.05 (d, *J* = 8.5 Hz, 4H), 7.75 (d, *J* = 8.7 Hz, 2H), 7.69 (d, *J* = 8.7 Hz, 2H), 7.34 (d, *J* = 7.6 Hz, 1H), 7.16 (t, *J* = 7.5, 7.4 Hz, 1H), 7.09 (t, *J* = 7.6, 7.3 Hz, 1H), 6.90 (d, *J* = 7.7 Hz, 1H), 3.67 (br s, 1H), 3.31 (d, *J* = 13.6 Hz, 1H), 3.00 (d, *J* = 13.6 Hz, 1H), δ 1.84 (s, 3H); ^13^C-NMR *δ* 164.0, 154.2, 148.6, 147.1, 144.9, 139.0, 137.4, 129.8, 127.9, 127.8, 127.0, 123.7, 123.6, 122.3, 121.5, 73.5, 43.1, 30.4; MS (EI) *m/z* (relative intensity) 402 (M^+^, 18), 387 (17), 280 (18), 239 (100), 193 (20); HRMS (EI) *m/z* calcd for C_22_H_18_N_4_O_4_ (M^+^) 402.1328, found 402.1323.

*2-Methyl-2,4-di-thiophen-2-yl-2,3-dihydro-1H-benzo[b][1,4]diazepine* (**3l**): ^1^H-NMR *δ* 7.39 (d, *J* = 4.8 Hz, 1H), 7.30–7.28 (m, 1H), 7.12–7.11 (m, 1H), 7.08–7.03 (m, 4H), 6.94–6.91 (m, 2H), 6.83–6.80 (m, 1H), 3.60 (br s, 1H, NH), 3.06 (d, *J* = 13.2 Hz, 1H), 3.00 (d, *J* = 13.2 Hz, 1H), 1.84 (s, 3H); ^13^C-NMR *δ* 162.4, 153.2, 137.2, 130.4, 128.6, 127.9, 127.6, 126.8, 126.3, 124.2, 122.8, 122.6, 122.0, 72.6, 44.4, 30.6; MS (EI) *m/z* (relative intensity) 324 (M^+^, 11), 201 (32), 200 (100), 109 (18); HRMS (EI) *m/z* calcd. for C_18_H_16_N_2_S_2_ (M^+^) 324.0755, found 324.0761.

*2,2,4-Trimethyl-2,3-dihydro-8-chloro-1H-1,5-benzodiazepine* (**3m**): ^1^H-NMR *δ* 7.07 (s, 1H), 7.00 (d, *J* = 8.4 Hz, 1H), 6.89–6.85 (m, 2H), 6.67 (s, 1H), 6.60 (d, *J* = 8.3 Hz, 1H), 3.02 (br s, 2H, NH), 2.30–2.29 (m, 6H), 2.19–2.16 (d, *J* = 12.3 Hz, 4H), 1.27 (s, 12H); ^13^C-NMR *δ* 173.8, 172.6, 141.4, 139.1, 138.4, 136.5, 130.0, 128.1, 126.4, 126.3, 125.1, 122.5, 121.3, 120.7, 68.2, 67.6, 45.1, 45.0, 30.4, 30.2, 29.64, 29.62; MS (EI) *m/z* (relative intensity) 222 (M^+^, 35), 209 (28), 207 (100), 167 (39), 166 (56); HRMS (EI) *m/z* calcd. for C_12_H_15_ClN_2_ (M^+^) 222.0924, found 222.0924.

*2-Methyl-2,4-diphenyl-8-chloro-2,3-dihydro-1H-1,5-benzodiazepine* (**3n**): ^1^H-NMR *δ* 7.60–7.56 (m, 8H), 7.35–7.18 (m, 14H), 7.05 (dd, *J* = 8.4, 2.4 Hz, 0.7H), 7.01–6.98 (dd, *J* = 8.4, 2.4 Hz, 1.3H), 6.85 (d, *J* = 2.4 Hz, 1.3H), 6.78 (d, *J* = 8.4 Hz, 0.7H), 3.62 (br s, 1.3H, NH), 3.52 (br s, 0.7H, NH), 3.17 (t, *J* = 13.4 Hz, 2H), 3.00–2.96 (m, 2H), 1.78 (s, 3H), 1.77 (s, 3H); ^13^C-NMR *δ* 168.7, 167.6, 147.1, 139.4, 139.1, 138.1, 131.0, 130.1, 130.1, 129.9, 128.4, 128.4, 128.1, 128.0, 127.2, 127.1, 127.0, 126.0, 125.3, 122.4, 121.3, 120.5, 73.7, 72.9, 43.2, 43.0, 30.0, 29.8; MS (EI) *m/z* (relative intensity) 346 (M^+^, 12), 230 (35), 229 (32), 228 (100), 167 (19), 103 (53); HRMS (EI) *m/z* calcd. for C_22_H_19_ClN_2_ (M^+^) 346.1237, found 346.1229.

*2,2,4,8-Tetramethyl-2,3-dihydro-1H-1,5-benzodiazepine* (**3o**): ^1^H-NMR *δ* 7.00 (d, *J* = 7.8 Hz, 1H), 6.93 (s, 1H), 6.77–6.75 (m, 2H), 6.62 (d, *J* = 7.8 Hz, 1H), 6.50 (s, 1H), 2.87 (br s, 1H, NH), 2.33 (s, 3H), 2.32 (s, 3H), 2.26 (s, 3H), 2.25 (s, 3H), 2.19 (s, 2H), 2.16 (s, 2H), 1.30 (s, 6H), 1.29 (s, 6H); ^13^C- NMR *δ* 172.4, 171.3, 140.9, 137.8, 137.7, 135.2, 135.1, 131.6, 126.9, 126.8, 126.0, 122.6, 121.9, 121.7, 68.3, 67.5, 45.1, 45.0, 30.4, 30.1, 29.7, 20.8, 20.5; MS (EI) *m/z* (relative intensity) 202 (M^+^, 30), 187 (100), 147 (23), 146 (38); HRMS (EI) *m/z* calcd. for C_13_H_18_N_2_ (M^+^) 202.1470, found 202.1469.

*2-Methyl-2,4-diphenyl-2,3-dihydro-8-methyl-1H-1,5-benzodiazepine* (**3p**): ^1^H-NMR *δ* 7.65–7.59 (m, 8H), 7.32–7.18 (m, 14H), 6.93 (d, *J* = 7.8 Hz , 0.8H), 6.88 (d, *J* = 7.8 Hz, 1.2H), 6.78 (d, *J* = 7.8 Hz, 0.8 H), 6.67 (s, 1.2H), 3.51 (br s, 2H, NH), 3.15 (t, *J* = 13.2 Hz, 2H), 2.98 (q, *J* = 7.2 Hz, 2H), 2.37 (d, *J* = 3.6 Hz, 6H), 1.77 (d, *J* = 4.2 Hz, 6H); ^13^C-NMR *δ* 167.8, 166.6, 147.7, 140.4, 139.8, 139.5, 137.9, 137.2, 136.2, 135.4, 131.2, 129.7, 129.5, 128.9, 128.6, 128.2, 128.0, 127.9, 127.0, 126.9, 125.4, 125.4, 125.3, 122.3, 121.4, 121.5, 73.8, 72.8, 43.3, 42.9, 29.9, 29.6, 21.0, 20.5; MS (EI) *m/z* (relative intensity) 326 (M^+^, 16), 311 (19), 209 (40), 208 (100), 207 (27), 200 (21), 103 (17), 77 (23); HRMS (EI) *m/z* calcd. for C_23_H_22_N_2_ (M^+^) 326.1783, found 326.1780.

*2,2,4,7,8-Pentamethyl-2,3-dihydro-1H-banzo[b][1,4]diazepine* (**3q**): ^1^H-NMR *δ* 6.92 (s, 1H), 6.52 (s, 1H), 2.34 (s, 3H), 2.20 (s, 3H), 2.19 (s, 3H), 2.18 (s, 3H), 1.32 (s, 6H); ^13^C-NMR *δ* 171.6, 138.5, 135.5, 133.7, 130.1, 127.9, 122.8, 67.9, 45.3, 30.4, 29.8, 19.2, 18.9; MS (EI) *m/z* (relative intensity) 216 (M^+^, 26), 201 (100), 161 (29), 160 (39), 145 (20); HRMS (EI) *m/z* calcd. for C_14_H_20_N_2_ (M^+^) 216.1626, found 216.1621.

*2,7,8-Trimethyl-2,4-diphenyl-2,3-dihydro-1H-benzo[b][1,4]diazepine* (**3r**): ^1^H-NMR *δ* 7.57 (t, *J* = 8.6 Hz, 4H), 7.23–7.12 (m, 7H), 6.62 (s, 1H), 3.40 (br s, 1H, NH), 3.09 (d, *J* = 8.7 Hz, 1H), 2.96 (d, *J* = 8.7 Hz, 1H), 2.24 (s, 6H), 1.73 (s, 3H); ^13^C-NMR *δ* 166.8, 147.8, 139.8, 137.7, 135.8, 134.8, 129.7, 129.6, 129.4, 128.2, 127.9, 126.9, 125.4, 122.3, 73.1, 43.3, 29.8, 18.4, 18.8; MS (EI) *m/z* (relative intensity) 340 (M^+^, 19), 325 (27), 223 (47), 222 (100); HRMS (EI) *m/z* calcd. for C_24_H_24_N_2_ (M^+^) 340.1939, found 340.1934.

*2,4-bis(4-Chlorophenyl)-2,7,8-trimethyl-2,3-dihydro-1H-benzo[b][1,4]diazepine* (**3s**): ^1^H-NMR *δ* 7.52–7.19 (m, 8H), 7.9 (s, 1H), 6.62 (s, 1H), 3.32 (br s, 1H, NH), 3.05 (d, *J* = 13.2 Hz, 1H), 2.87 (d, *J* = 13.2 Hz, 1H), 2.24 (s, 6H), 1.71 (s, 3H); ^13^C-NMR *δ* 165.2, 146.1, 138.1, 137.1, 135.8, 135.4, 135.3, 132.9, 130.0, 129.7, 128.4, 128.3, 128.2, 127.1, 122.4, 73.0, 43.1, 29.8, 19.4, 18.8; MS (EI) *m/z* (relative intensity) 408 (M^+^, 3), 305 (12), 304 (70), 289 (100), 131 (18); HRMS (EI) *m/z* calcd. for C_24_H_22_N_2_Cl_2_ (M^+^) 408.1160, found 408.1155.

*7,8-Dichloro-2,2,4-trimethyl-2,3-dihydro-1H-benzo[b][1,4]diazepine* (**3t**): ^1^H-NMR *δ* 7.20 (s, 1H), 6.81 (s, 1H), 3.07 (br s, 1H, NH), 2.34 (s, 3H), 2.25 (s, 2H), 1.33 (s, 6H); ^13^C-NMR *δ* 173.9, 139.7, 137.7, 128.3, 128.1, 124.4, 122.2, 67.7, 45.3, 30.5, 29.9; MS (EI) *m/z* (relative intensity) 256 (M^+^, 28), 258 (18), 243 (58), 241 (100), 203 (25), 202 (37), 201 (46), 200 (58); HRMS (EI) *m/z* calcd. for C_12_H_14_N_2_Cl_2_ (M^+^) 256.0534, found 256.0529.

*(E)-7,8-Dichloro-2-methyl-2,4-diphenyl-2,3-dihydro-1H-benzo[b][1.4]diazepine* (**3u**): ^1^H-NMR *δ* 7.54 (t, *J* = 6.8, 6.6 Hz, 4H), 7.40 (s, 1H), 7.33-7.17 (m, 6H), 6.92 (s, 1H), 3.59 (br s, 1H, NH), 3.17 (d, *J* = 13.4 Hz, 1H), 2.97 (d, *J* = 13.4 Hz, 1H), 1.75 (s, 3H), 1.57 (s, 1H); ^13^C-NMR *δ* 168.8, 146.8, 139.2, 138.9, 137.7, 130.2, 129.9, 128.8, 128.1, 127.1, 125.3, 124.1, 121.8, 72.9, 43.2, 29.9; MS (EI) *m/z* (relative intensity) 380 (M^+^, 8), 260 (10), 265 (19), 264 (61), 262 (100), 103 (23), 77 (12); HRMS (EI) *m/z* calcd. for C_22_H_18_N_2_Cl_2_ (M^+^) 380.0874, found 380.0842.

*2,2,4-Trimethyl-2,3-dihydro-1H-naphtho[2,3-b][1,4]diazepine* (**3v**): ^1^H-NMR *δ* 7.72 (d, *J* = 7.8 Hz, 1H), 7.61 (d, *J* = 7.9 Hz, 1H), 7.55 (s, 1H), 7.33-7.29 (m, 2H), 7.08 (s, 1H), 2.38 (s, 3H), 2.17 (s, 2H), 1.33 (br s, 1H, NH); ^13^C-NMR *δ* 173.1, 141.9, 137.6, 131.9, 130.3, 127.4, 125.8, 125.2, 124.0, 123.6, 117.6, 65.9, 44.6, 29.9, 29.6; MS (EI) *m/z* (relative intensity) 238 (M^+^, 25), 224 (18), 223 (100), 183 (42), 182 (37), 115 (17); HRMS (EI) *m/z* calcd. for C_16_H_18_N_2_ (M^+^) 238.1470, found 238.1465.

*2,2',4,4,4',4'-Hexamethyl-4,4',5,5'-tetrahydro-3H,3'H-7,7'-dibenzo[b][1,4]diazepine* (**3w**): mixture of two isomers; ^1^H-NMR *δ* 7.39 (s, 1H), 7.20 (m, 3H), 6.92 (d, 1H, *J* = 9.7 Hz), 6.74 (d, 1H, *J* = 5.0 Hz) 1H), 3.01 (br s, 2H), 2.36 (s, 6H), 2.56 (s, 4H), 1.32 (s, 12H); ^13C^-NMR *δ* 172.8, 172.7, 172.5, 172.4, 138.4, 138.3, 138.1, 138.0, 137.5, 136.9, 134.4, 127.5, 127.4, 125.3, 124.8, 123.9, 123.7, 122.1, 122.0, 120.5, 120.2, 119.8, 119.6, 68.1, 67.9, 45.4, 45.3, 30.7, 30.6, 29.9, 29.8. MS (EI) *m/z* (relative intensity) 374 (M^+^, 40), 359 (100), 319 (23), 263 (7).
